# *Shigella* Bacteremia, Georgia, USA, 2002–2012^1^

**DOI:** 10.3201/eid2601.181698

**Published:** 2020-01

**Authors:** Melissa Tobin-D’Angelo, Nadine Oosmanally, Siri N. Wilson, Evan J. Anderson, Suzanne Segler, Lynett Poventud

**Affiliations:** Georgia Department of Public Health, Atlanta, Georgia, USA (M. Tobin-D’Angelo, N. Oosmanally, S.N. Wilson, L. Poventud);; Georgia Emerging Infections Program, Atlanta (M. Tobin-D’Angelo, N. Oosmanally, E.J. Anderson, S. Segler);; Emory University School of Medicine, Atlanta (E.J. Anderson);; Veterans Administration Medical Center, Atlanta (S. Segler)

**Keywords:** Shigella, bacteremia, HIV/AIDS, Georgia, United States, bacteria, surveillance, HIV

## Abstract

*Shigella* commonly causes gastroenteritis but rarely spreads to the blood. During 2002–2012, we identified 11,262 *Shigella* infections through population-based active surveillance in Georgia; 72 (0.64%) were isolated from blood. Bacteremia was associated with age >18 years, black race, and *S. flexneri*. More than half of patients with bacteremia were HIV-infected.

*Shigella* is among the more common bacterial causes of diarrhea. In the United States, ≈500,000 illnesses occur annually, and prevalence ranges from 3.8 to 5 cases per 100,000 population (*1*,*2*). Transmission occurs not only through contaminated food and water but also through fecal–oral transmission; ingestion of only 10–100 *Shigella* organisms can cause disease (*1*,*3*). Risk factors for *Shigella* include daycare attendance, international travel, and men having sex with men (*1*,*4*). Although 4 main serogroups exist, *S. sonnei* accounts for ≈70% of US isolates, and *S. flexneri* accounts for ≈24% (*1*).

*Shigella* bacteremia is uncommon, and risk factors are not well described in the United States. Internationally, many patients with *Shigella* bacteremia are HIV infected (*5*,*6*). In the United States, case series describe *Shigella* bacteremia in children <1 year of age and in adults with malnutrition, HIV infection, and other immunocompromising conditions (e.g., diabetes mellitus, malignancy) (*7*,*8*). Data collected by the Georgia Department of Public Health as part of the Foodborne Disease Active Surveillance Network (FoodNet) provided an opportunity to better understand the epidemiology of *Shigella* bacteremia.

## The Study

The Georgia Department of Public Health participates in FoodNet as part of the Emerging Infections Program, a collaboration between 10 US sites and the Centers for Disease Control and Prevention. FoodNet conducts active surveillance for laboratory-confirmed infections of 7 bacterial and 2 parasitic pathogens commonly transmitted through food (*1*). Georgia residents who had laboratory-confirmed *Shigella* were identified through FoodNet surveillance data during 2002–2012. We excluded duplicates, defined as an additional positive laboratory test within 30 days of the original diagnosis with the same *Shigella* serogroup. We also excluded isolates from nonblood and nonfecal sources or unknown sources from the analyses. In addition, after initial case-counts, we excluded *S. boydii* and *S. dysenteriae* because of small numbers. For patients with *Shigella*, we obtained HIV status from the Georgia Department of Public Health HIV surveillance data collected in the Enhanced HIV/AIDS Reporting System, which included the year of diagnosis, AIDS status, and route of HIV transmission (if known). We compared case-patient information between patients with fecal versus blood isolates and described factors associated with HIV among patients with bacteremia. We used ArcView GIS version 10.3 (https://www.esri.com) to characterize case-patients by county.

The Georgia Public Health Laboratory performed *Shigella* identification, serogroup determination, serotyping, and pulse-field gel electrophoresis. Detailed serogroup and serotype data were available for 2005–2012.

We analyzed data using SAS version 9.3 (https://www.sas.com). We created logistic regression models using significant variables and possible confounders and identified the final model using Score Selection.

For 2002–2012, we identified 11,262 *Shigella* infections among Georgia residents. During this time, 10,806 (96.0%) of cultures were isolated from feces, and 72 (0.66%) were isolated from blood. We excluded 13 *S. dysentariae* (1 blood isolate) and 31 *S. boydii* (all fecal isolates) from further analysis. Patients with *Shigella* bacteremia were concentrated in the Atlanta metropolitan area (Table). Fifty-three (74%) blood isolates versus 3,089 (29%) fecal isolates were from patients >18 years of age. No bacteremia cases were outbreak-associated. Only 1 (0.87%) of 114 patients with documented international travel had bacteremia. Demographic variables significantly associated with bacteremia on bivariate analysis included male sex, black race, and residence in the Atlanta metropolitan area. Analysis of clinical variables demonstrated that patients with bacteremia were more likely to be hospitalized (61% vs. 17%; p<0.001) and to die (Table). Male sex, age >18 years, and *S. flexneri* serotypes remained significant on multivariate analysis.

**Table:** Epidemiologic and clinical characteristics of shigellosis cases, Georgia, USA, 2002–2012

Characteristic	Isolates, no. postive/total no. (%)*				Odds ratio (95% CI)	
	All *Shigella*	Feces	Blood		Univariate†	Multivariate‡
Outbreak-associated case‡	591/3,681 (16.1)	586/3,509 (16.7)	0/38		15.46 (0.95–251.97)	
International travel–acquired case§	114/3,681 (8.3)	113/1,315 (8.6)	1/17 (5.9)		1.51 (0.20−11.5)	
Hospitalized patient	1,940/10,443 (18.6)	1,826/10,804 (16.9)	44/72 (61.1)		0.10 (0.06–0.18)	
Died	35/11,262 (0.31)	29/8,823 (0.3)	2/66 (3.0)		0.11 (0.25–0.45)	
Atlanta resident¶	3,793/11,262 (33.7)	3,647/10,794 (33.8)	50/72 (69.4)		4.46 (2.70–7.38)	
Patient race/ethnicity						
White	4,396/9,502 (46.3)	4,782/9,026 (53.0)	12/71 (16.9)		7.36 (4.10– 13.20)	
Other	484/9,502 (5.1)	364/9,026 (4.0)	1/71 (1.4)		6.50 (0.40– 105.15)	
Hispanic	874/7,545 (11.6)	844/6,405 (13.2)	1/63 (1.6)		8.18 (1.13– 59.04)	
Black	4,082/9,502 (43.0)	3,880/9,026 (43.0)	58/71 (81.7)		6.03 (3.30–11.03)	5.40 (2.94– 9.89)
Male sex	5,500/11,196 (49.1)	5,348/10,744 (49.8)	51/72 (70.8)		2.45 (1.47–4.08)	1.50 (0.83– 2.68)
Age >18 y	3,321/11,262 (29.5)	3,089/10,773 (28.7)	53/72 (73.6)		6.83 (4.04–11.56)	4.61 (2.55– 8.34)
*S. flexneri* serogroup§	755/6,960 (10.8)	710/6,742 (10.5)	26/52 (50.0)		8.50 (4.91–14.72)	1.96 (1.06– 3.61)

*Unknown results were excluded. †Odds ratio in comparison to isolation from feces. Blank cells indicate variables were not included in the final model. ‡Final logistic regression model results (cases with missing data were excluded from the multivariate analysis). §Reported only for 2005–2012. ¶Atlanta resident is a patient residing in the 20-county metropolitan statistical area.

Thirty-seven (51%) of the 72 patients with bacteremia were known to be HIV-infected. All but 3 HIV-infected patients resided in the Atlanta metropolitan area. Among those known to be HIV-infected, 92% were black, 97% had a known AIDS diagnosis, 97% were male, and 68% were known to be men who have sex with men (MSM), a risk factor for HIV acquisition.

Among *Shigella* fecal isolates typed from 2005 to 2012, *S. sonnei* predominated (6,017 [89%] vs. 710 [10%] *S. flexneri*). In contrast, equal numbers of *S. sonnei* and *S. flexneri* (26 cases each) were identified from blood (p<0.01 for the difference between proportions in the blood vs. feces). *S. flexneri* serotype 2a comprised 5 isolates, serotype 3 comprised 6 isolates, and serotype 4a comprised 9 isolates, for a total of 85% of these 26 isolates. No serogroup trend over time was apparent with *S. flexneri*; the number of bacteremia cases ranged from 1 to 8 per year, peaking in 2009, with a single case each in 2002, 2006, and 2008. For *S. sonnei*, 2–6 bacteremia cases were identified per year, peaking in 2003, with a single case each in 2009 and 2010. Among bacteremia patients with serogroup determination, factors associated with HIV infection were black race (odds ratio 5.2 [95% CI 1.3–20.6]) and *S. flexneri* infection (odds ratio 40.4 [95% CI 8.0–204.9]).

## Conclusions

The relative predominance of *S. flexneri* among blood isolates in comparison to fecal isolates in this analysis is noteworthy. In other large international series, *S. flexneri* has been identified in most bacteremia patients (*5*,*8*,*9*). More than half of the bacteremia patients in our study were known to be HIV-infected. Other researchers have noted the prominence of *S. flexneri* among MSM and HIV-infected persons (*1*,*4*,*10*,*11*). In data from South Africa, *S. flexneri* serotype 2a was also identified in 30% of invasive isolates (*6*). It is unclear whether these serotypes might have increased virulence or might be more common because of transmission networks, particularly among the HIV-infected patients in this study. It is clear, however, that HIV infection correlates with the epidemiology of *Shigella* bacteremia, particularly in the Atlanta area. Some of the demographic factors associated with bacteremia (e.g., black race, identification of *S. flexneri*) also were associated with HIV infection within the subset of patients with bacteremia. The predominance of the MSM risk factor among HIV-infected patients, along with the low infectious dose and possibility of sexual transmission of *Shigella*, make it possible that we could be missing outbreaks within this population (*7*). Clinicians caring for HIV-infected patients should be aware of the possibility of *Shigella* bacteremia. Additionally, identification of *Shigella* bacteremia in an adult should prompt HIV testing unless another immunocompromising condition exists.

Limitations of our study include the unavailability of epidemiologic and clinical data for all patients in the study. We had information about HIV status only for patients with *Shigella* bacteremia. Other clinical characteristics that might be associated with *Shigella* bacteremia were not collected and could not be analyzed (e.g., malignancy, transplantation) (*4**,**10*). Finally, some epidemiologic information and detailed identification of *Shigella* serogroups and serotypes was not available until 2005.

In summary, although *S. sonnei* predominated among fecal isolates in this study, similar numbers of *S. sonnei* and *S. flexneri* were identified in blood cultures. *Shigella* bacteremia, particularly when caused by *S. flexneri*, should prompt evaluation for a concomitant HIV infection among certain adult populations.

## References

[1] Shiferaw B, Shallow S, Marcus R, Segler S, Soderlund D, Hardnett FP, et al.; Emerging Infections Program FoodNet Working Group. Trends in population-based active surveillance for shigellosis and demographic variability in FoodNet sites, 1996-1999. Clin Infect Dis. 2004;38(Suppl 3):S175–80. 10.1086/381584 15095187

[2] Centers for Disease Control and Prevention (CDC). Vital signs: incidence and trends of infection with pathogens transmitted commonly through food—foodborne diseases active surveillance network, 10 U.S. sites, 1996-2010. MMWR Morb Mortal Wkly Rep. 2011;60:749–55. 21659984

[3] DuPont HL, Levine MM, Hornick RB, Formal SB. Inoculum size in shigellosis and implications for expected mode of transmission. J Infect Dis. 1989;159:1126–8. 10.1093/infdis/159.6.1126 2656880

[4] Tauxe RV, McDonald RC, Hargrett-Bean N, Blake PA. The persistence of *Shigella flexneri* in the United States: increasing role of adult males. Am J Public Health. 1988;78:1432–5. 10.2105/AJPH.78.11.1432 3052115 PMC1350234

[5] Davies NE, Karstaedt AS. *Shigella bacteraemia* over a decade in Soweto, South Africa. Trans R Soc Trop Med Hyg. 2008;102:1269–73. 10.1016/j.trstmh.2008.04.037 18550134

[6] Keddy KH, Sooka A, Crowther-Gibson P, Quan V, Meiring S, Cohen C, et al.; Group for Enteric, Respiratory, and Meningeal Disease Surveillance in South Africa (GERMS-SA). Systemic shigellosis in South Africa. Clin Infect Dis. 2012;54:1448–54. 10.1093/cid/cis224 22474223

[7] Hawkins C, Taiwo B, Bolon M, Julka K, Adewole A, Stosor V. *Shigella sonnei* bacteremia: two adult cases and review of the literature. Scand J Infect Dis. 2007;39:170–3. 10.1080/00365540600786580 17366038

[8] Struelens MJ, Patte D, Kabir I, Salam A, Nath SK, Butler T. *Shigella* septicemia: prevalence, presentation, risk factors, and outcome. J Infect Dis. 1985;152:784–90. 10.1093/infdis/152.4.784 4045231

[9] Greenberg D, Marcu S, Melamed R, Lifshitz M. *Shigella* bacteremia: a retrospective study. Clin Pediatr (Phila). 2003;42:411–5. 10.1177/000992280304200504 12862343

[10] Aragón TJ, Vugia DJ, Shallow S, Samuel MC, Reingold A, Angulo FJ, et al. Case-control study of shigellosis in San Francisco: the role of sexual transmission and HIV infection. Clin Infect Dis. 2007;44:327–34. 10.1086/510593 17205436

[11] Baer JT, Vugia DJ, Reingold AL, Aragon T, Angulo FJ, Bradford WZ. HIV infection as a risk factor for shigellosis. Emerg Infect Dis. 1999;5:820–3. 10.3201/eid0506.990614 10603219 PMC2640795

